# Effects of long-term anti-seizure medication monotherapy on all-cause death in patients with post-stroke epilepsy: a nationwide population-based study in Taiwan

**DOI:** 10.1186/s12883-021-02241-5

**Published:** 2021-06-21

**Authors:** Chia-Yu Hsu, Chun-Yu Cheng, Jiann-Der Lee, Meng Lee, Bruce Ovbiagele

**Affiliations:** 1 Departments of Neurology, Chang Gung University College of Medicine, Chang Gung Memorial Hospital, Chiayi, Taiwan; 2 Departments of Neurosurgery, Chang Gung University College of Medicine, Chang Gung Memorial Hospital, Chiayi, Taiwan; 3 Department of Neurology, University of California, California San Francisco, USA

**Keywords:** Epilepsy, Ischemic stroke, Anticonvulsants, Death

## Abstract

**Objective:**

We aim to compare the effect of long-term anti-seizure medication (ASM) monotherapy on the risk of death and new ischemic stroke in patients with post-stroke epilepsy (PSE).

**Patients and methods:**

We identified all hospitalized patients (≥ 20 years) with a primary diagnosis of ischemic or hemorrhagic stroke from 2001 to 2012 using the National Health Insurance Research Database in Taiwan. The PSE cohort were defined as the stroke patients (1) who had no epilepsy and no ASMs use before the index stroke, and (2) who had epilepsy and ASMs use after 14 days from the stroke onset. The patients with PSE receiving ASM monotherapy were enrolled and were categorized into phenytoin, valproic acid, carbamazepine, and new ASM groups. We employed the Cox regression model to estimate the unadjusted and adjusted hazard ratios (HRs) with 95 % confidence intervals (CIs) of death and new ischemic stroke within 5 years across all groups, using the new ASM group as the reference.

**Results:**

Of 6962 patients with PSE using ASM monotherapy, 3917 (56 %) were on phenytoin, 1623 (23 %) on valproic acid, 457 (7 %) on carbamazepine, and 965 (14 %) on new ASMs. After adjusting for confounders, compared with new ASM users, phenytoin users had a higher risk of death in 5 years (HR: 1.64; 95 % CI: 1.06–2.55). On the other hand, all ASM groups showed a similar risk of new ischemic stroke in 5 years.

**Conclusions:**

Among patients with PSE on first-line monotherapy, compared to new ASMs, use of phenytoin was associated with a higher risk of death in 5 years.

**Supplementary Information:**

The online version contains supplementary material available at 10.1186/s12883-021-02241-5.

## Introduction

Cerebrovascular disease is a common underlying mechanism for late-onset epilepsy [1]. Long term cumulative risk of post-stroke epilepsy (PSE) after a cerebrovascular event varies from 2 to 15 % according to different definitions of PSE, stroke types, and follow-up durations [2]. Anti-seizure medications (ASMs) are often administered to prevent recurrent seizures in patients with PSE [3]. The choice of first-line ASMs in patients with PSE are diverse. International League Against Epilepsy had reported that carbamazepine, phenytoin, levetiracetam and zonisamide had level A evidence of efficacy for adults with focal seizures; gabapentin and lamotrigine had level A evidence of efficacy for elderly adults with focal seizures [4]. Two small randomized controlled trials in patients with PSE showed that lamotrigine and levetiracetam were effective for seizure control and were better tolerated compared to carbamazepine [5–7]. In general, more than 70 % of patients with PSE can be well-controlled under ASM monotherapy [6–8].

Except for seizure control effectiveness, the effects of ASMs on cardiovascular outcomes and mortality are also important issues. A nationwide cohort study in Denmark found that ASM-treated epilepsy was associated with a higher risk of myocardial infarction, stroke, cardiovascular death and all-cause death [9]. A range of ASMs, especially first-generation of ASMs, have been linked to atherothrombotic risk factors, such as increased level of homocysteine, hyperlipidemia, weight gain, and insulin resistance [9]. One study had found that phenytoin was associated with an increased stroke risk compared to carbamazepine in patients with epilepsy [10]. It has been reported that stroke patients with PSE have higher mortality and worse functional outcome compared to those without PSE [11–13]. Therefore, the impact of ASMs on the risk of all-cause death and recurrent stroke should be particularly considered. However, studies about this issue are scarce and diverse. Therefore, we conducted this nationwide cohort study to evaluate the comparative effects of long-term ASM monotherapy on the risks of all-cause death and new ischemic stroke in patients with PSE.

## Methods

### Data source

We conducted a nationwide retrospective cohort study using the National Health Insurance Research Database (NHIRD). The Taiwan National Health Insurance program was launched in 1995. It covers 99 % of the population and reimburses for outpatient and inpatient services as well as prescription drugs. In the study period, the diagnosis code in NHIRD consists of the 9th revision of the International Classification of Diseases (ICD-9). The diagnoses of acute ischemic stroke and epilepsy have been validated in NHIRD [14–16]. This study has been approved by the institutional review board of Chang Gung Memorial Hospital, Chiayi, Taiwan.

### Study Population: defining the patients with PSE (Fig. 1)

We identified all hospitalized patients (≥ 20 years) who were admitted with a primary diagnosis of ischemic or hemorrhagic stroke (ICD-9 codes 433.X1, 434.X1, 436, 431) encountered between January 1, 2001 and December 31, 2012. Hospitalizations due to transient ischemic stroke (ICD-9 435.9) or subarachnoid hemorrhage (ICD-9 430) were not included. For each case, the first stroke episode during the study period was defined as the index stroke. We excluded (1) patients without computed tomography or magnetic resonance imaging of brain during the index stroke or within the 3 days before the index stroke (because the diagnosis of stroke may be indefinite), (2) patients with an epilepsy diagnosis (ICD-9 345.00) before the index stroke, (3) patients who had used any ASMs within 180 days before the index stroke, and (4) patients who died within 2 months after the index stroke (because the early mortality after the stroke is less likely related to ASMs use).

PSE was defined as presence of epilepsy diagnosis (ICD-9 345.00) (once in inpatient or twice in outpatient diagnosis) along with usage of oral ASM for more than 28 days. We extracted data of ASMs usage after 14 days from the index stroke, because ASMs used within 14 days from the index stroke may be used for early seizure control or prevention. ASMs in this study included carbamazepine, clobazam, gabapentin, lacosamide, lamotrigine, levetiracetam, oxcarbazepine, perampanel, phenobarbital, phenytoin, pregabalin, rufinamide, valproic acid, tiagabine, topiramate, vigabatrin, and zonisamide. Intravenous ASMs were not included in our study because we focused on the long-term effects of ASMs.

This was a nationwide study that included all available and eligible patients. The requirement of informed consent from subjects included in this study was waived.

### Grouping of anti-seizure medications (Fig. 1)

Among the patients with PSE, we identified the patients whose first-line treatment was a single ASM for more than 28 days. These patients were grouped according to the first-line ASM they used, including phenytoin group, valproic acid group, carbamazepine group and new ASM (including monotherapy with clobazam, gabapentin, lacosamide, lamotrigine, levetiracetam, oxcarbazepine, perampanel, pregabalin, rufinamide, tiagabine, topiramate, vigabatrin, and zonisamide) group. We excluded patients who used phenobarbital as the first ASM because the case number was too small. Any gap between ASMs prescription more than 28 days was considered as discontinuing of the ASM.

### Study design

This was a retrospective cohort study. For each patient, the first date of the ASM use was defined as index date of ASM use. Each patient was followed from the index date of ASM use until one of the following events happened: (1) death from any cause; (2) end of the database (December 31, 2013); (3) switching to or adding on another ASMs; (4) discontinuing the first ASM or (5) follow-up for 5 years. The primary study outcome was all-cause death and the secondary study outcome was new ischemic stroke in the follow-up period. Death was defined by death mark in NHIRD, and new ischemic stroke was defined by new hospitalization with a primary diagnosis of ischemic stroke (ICD-9 codes 433.X1, 434.X1, 436) with brain imaging during or within the 3 days before the hospitalization.

Comorbidity information was extracted by ICD-9 codes before the index date of ASM use for each case, including hypertension (ICD-9 401–405), ischemic heart disease (ICD-9 410–414), diabetes mellitus (ICD-9 250), hyperlipidemia (ICD-9 272), atrial fibrillation (ICD-9 427.31), heart failure (ICD-9 428), chronic kidney disease (ICD-9 585, 403), peripheral vascular disease (ICD-9 443.9), mood disorders (ICD-9 296, 311), dementia (ICD-9 290), migraine (ICD-9 346) and prior stroke (before the index stroke) (ICD-9 430–434, 436–438). Stroke severity of the index stroke was evaluated by the stroke severity index (airway suctioning, bacterial sensitivity test, general ward stays, intensive care unit stay, nasogastric intubation, osmotherapy, and urinary catheterization), developed specifically to evaluate the severity of strokes in Taiwan NHIRD [17, 18]. Information about concomitant medication use (including antiplatelets, anticoagulants, antidepressants and statin) from the index date of ASM use to the end of the follow-up period was extracted for each case.

### Statistical analysis

The differences among the baseline characteristics of the four ASM groups were analyzed by Analysis of Variance (ANOVA) for normally distributed variables, and Pearson’s Chi Square test for categorical variables.

We employed the Cox regression model to estimate the unadjusted and adjusted hazard ratios (HRs) and 95 % confidence intervals (CIs) of the relative risk of death and new ischemic stroke of the phenytoin group, valproic acid group and carbamazepine group, using the new ASM group as the reference. The relative risk of new ischemic stroke in the four ASM groups were analyzed by competing risk analysis with death as the competing risk. The model was adjusted for age, gender, index stroke types, all of the above-mentioned comorbidities, concomitant medications use (anticoagulants, antidepressants, antiplatelet agents, and statin), and year of index date of ASM use. For the groups which had significantly different risk of death or new ischemic stroke in the follow-up period compared to the reference group, we further did subgroup analyses. Subgroup analyses were performed to assess the interaction effect between different patient characteristics and ASMs use. We tested the significance of the interaction terms in the regression models using the Wald test for interactions.

To evaluate the impact of ASM switches, we performed a sensitivity analysis called the “first ASM model”. In this model, patients were classified according to the initial ASM they received, regardless of subsequent usage pattern.

Statistical significance was determined using 95 % CIs or a *P* value < 0.05. Statistical analysis was performed using the SAS statistical package (release 9.4, SAS Institute Inc, Cary, NC).

## Results

**Fig. 1:**
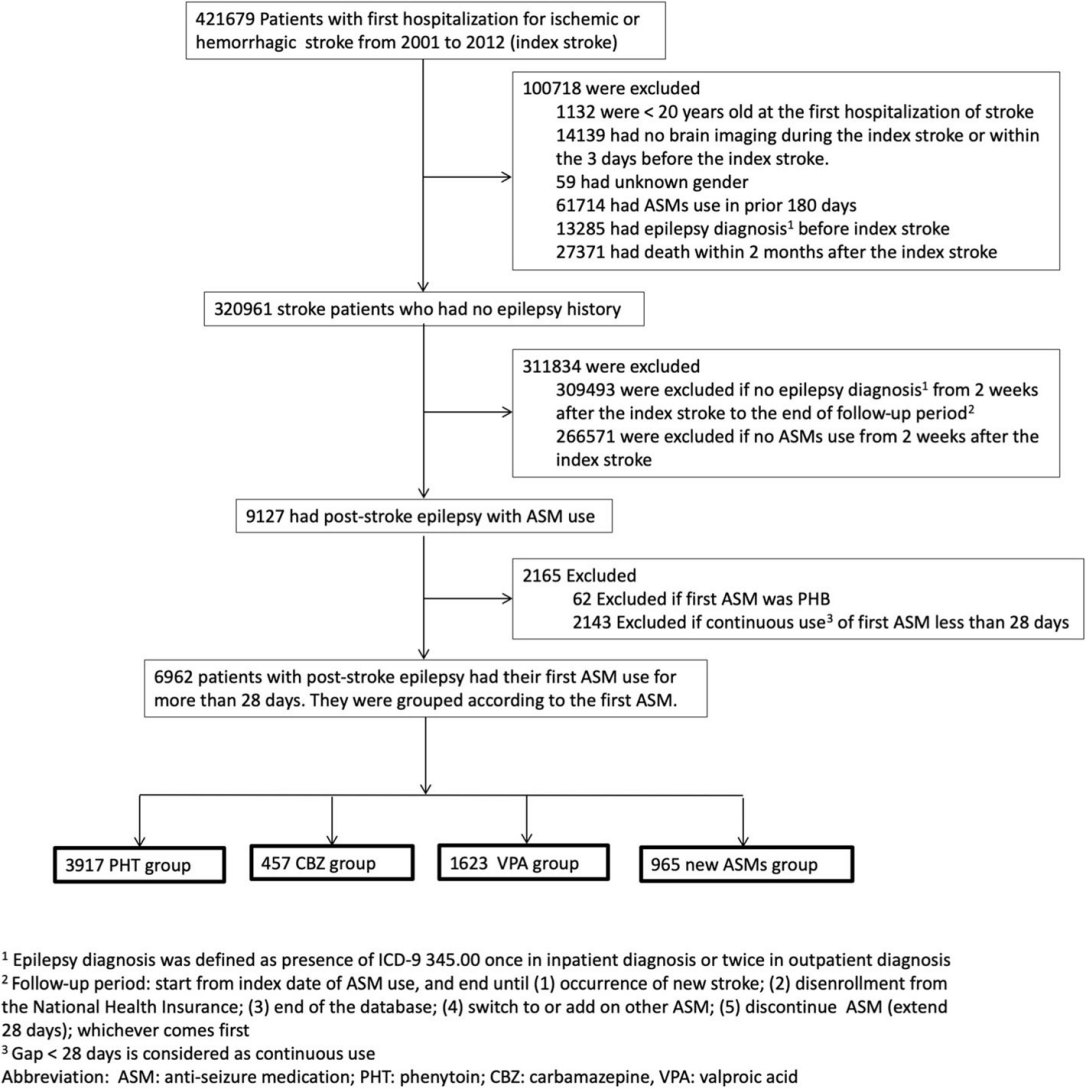
Flow chart of study cohort selection and grouping

In the study period, 320,961 stroke patients without previous epilepsy history or previous ASMs use were identified, and 9127 (2.8 %) of them had PSE with ASM use. Among these patients, 6962 cases received the first ASM monotherapy continuously for more than 28 days, and they were classified according to the first ASM they received: 3917 in phenytoin group, 1623 in valproic acid group, 457 in carbamazepine group, and 965 in new ASM group. (Fig. 1) In the new ASMs group, 307 (31.8 %) used gabapentin, 262 (27.2 %) used levetiracetam, 259 (26.8 %) used oxcarbazepine, 71 (7.4 %) used topiramate, 39 (4 %) used lamotrigine, 12 (1.2 %) used clobazam, 8 (0.8 %) used pregabalin and 7 (0.7 %) used vigabatrin. For the subtypes of the index stroke, 4650 (66.8 %) had ischemic stroke and 2312 (33.2 %) had hemorrhagic stroke. Patient characteristics of the four ASM groups were shown in Table 1. The mean age of patients in the phenytoin, valproic acid, carbamazepine and new ASM groups was 67.6, 68.7, 66.3 and 66.1 years, respectively. The mean latency from the index stroke to the PSE in the phenytoin, valproic acid, carbamazepine and new ASM groups were 1.5, 1.6, 1.0, and 1.7 years, respectively. The index stroke subtype was hemorrhagic stroke in 1420 (36.3 %), 456 (28.1 %), 169 (37 %) and 267 (27.7 %) patients in the phenytoin, valproic acid, carbamazepine and new ASM groups, respectively. (Table 1)

**Table 1:** Basic characteristics of patients with post-stroke epilepsy in the four ASM groups

	Phenytoin (n = 3917)	Valproic acid (n = 1623)	Carbamazepine (n = 457)	New ASM (n = 965)
Age of ASM use, y, mean(SD)	67.6 (14.0)	68.7 (14.4)	66.3 (13.2)	66.1 (13.7)
Hemorrhagic stroke, n (%)	1420 (36.3 %)	456 (28.1 %)	169 (37.0 %)	267 (27.7 %)
Male, n (%)	2525 (64.5 %)	998 (61.5 %)	248 (54.3 %)	587 (60.8 %)
Hypertension, n (%)	3558 (90.8 %)	1468 (90.5 %)	422 (92.3 %)	863 (89.4 %)
Diabetic mellitus, n (%)	1609 (41.1 %)	668 (41.2 %)	194 (42.5 %)	420 (43.5 %)
Hyperlipidemia, n (%)	1608 (41.1 %)	761 (46.9 %)	205 (44.9 %)	534 (55.3 %)
Ischemic heart disease, n (%)	1780 (45.4 %)	779 (48.0 %)	183 (40.0 %)	430 (44.6 %)
Heart failure, n (%)	524 (13.4 %)	228 (14.1 %)	45 (9.9 %)	97 (10.1 %)
Atrial fibrillation, n (%)	767 (19.6 %)	355 (21.9 %)	40 (8.8 %)	166 (17.2 %)
Prior stroke, n (%)	1125 (28.7 %)	499 (30.8 %)	120 (26.3 %)	269 (27.9 %)
Chronic kidney disease, n (%)	345 (8.8 %)	143 (8.8 %)	41 (9.0 %)	110 (11.4 %)
Peripheral vascular disease, n (%)	20 (4.4 %)	123 (3.1 %)	72 (4.4 %)	46 (4.8 %)
Mood disorder, n (%)	352 (9.0 %)	210 (12.9 %)	50 (10.9 %)	99 (10.3 %)
Dementia, n (%)	719 (18.4 %)	354 (21.8 %)	60 (13.1 %)	115 (11.9 %)
Migraine, n (%)	128 (3.3 %)	63 (3.9 %)	24 (5.3 %)	54 (5.6 %)
Stroke severity index				
Mild, n (%)	515 (13.2 %)	312 (19.2 %)	74 (16.2 %)	188 (19.5 %)
Moderate, n (%)	2573 (65.7 %)	1014 (62.5 %)	348 (76.2 %)	632 (65.5 %)
Severe, n (%)	829 (21.2 %)	297 (18.3 %)	35 (7.7 %)	145 (15.0 %)
Concomitant medications				
Anticoagulants, n (%)	241 (6.2 %)	163 (10.0 %)	16 (3.5 %)	95 (9.8 %)
Antidepressants, n (%)	545 (13.9 %)	305 (18.8 %)	120 (26.3 %)	230 (23.8 %)
Antiplatelet, n (%)	1900 (48.5 %)	835 (51.5 %)	242 (53.0 %)	513 (53.2 %)
Statins, n (%)	500 (12.8 %)	249 (15.3 %)	71 (15.5 %)	213 (22.1 %)
Interval from index stroke to index date of ASM use, years, mean(SD)	1.5 (1.7)	1.6 (1.8)	1.0 (1.1)	1.7 (2.0)

Abbreviations: *ASM* anti-seizure medication; *SD* standard deviation

**Fig. 2:**
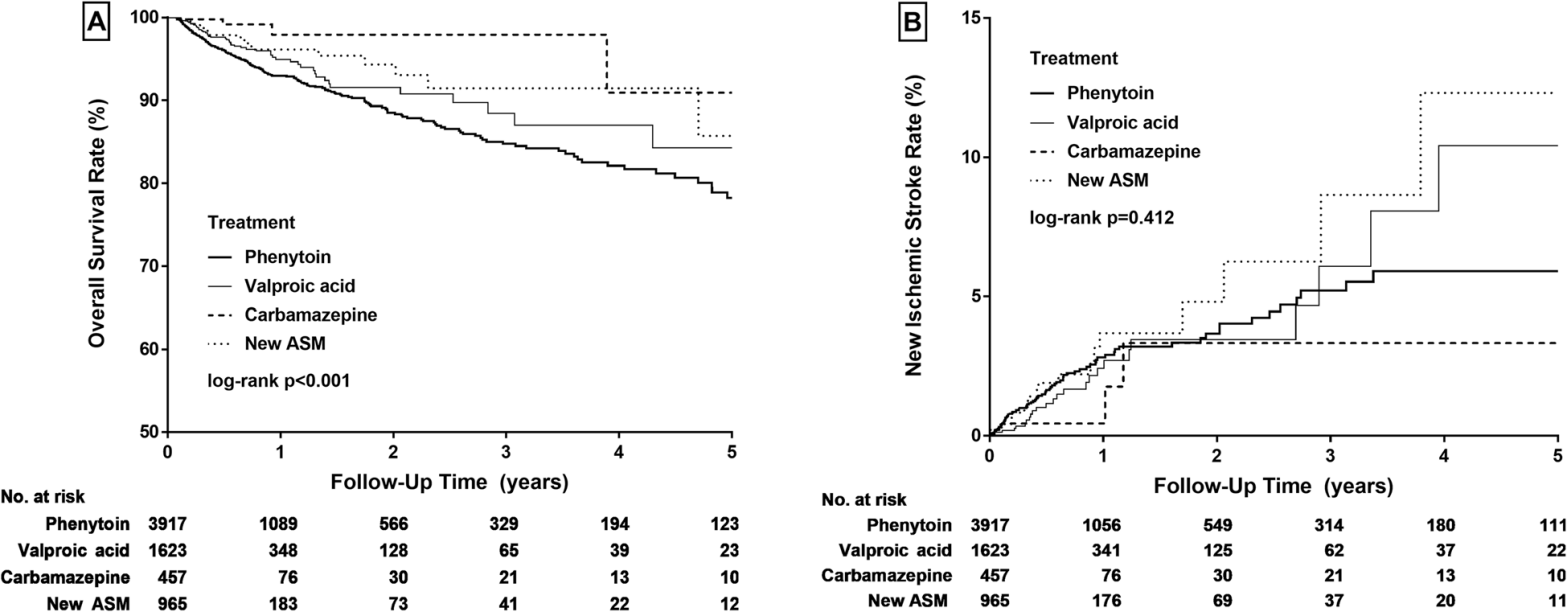
The Kaplan–Meyer curves of survival rate (**a**), and new ischemic stroke rate (**b**)

The number of death or new ischemic stroke in the follow-up period and the mean interval from the date of ASM use to the date of death or new ischemic stroke in the four ASM groups were shown in Table 2. Compared to the new ASM group, the risk of the 5-year mortality was higher in phenytoin group (HR 1.64, 95 % CI 1.06–2.55) but was not different in valproic acid group (HR 1.03, 95 % CI 0.62–1.70) and carbamazepine group (HR 0.47, 95 % CI 0.14–1.62), after adjustment of covariates. The risk of the new ischemic stroke was not different in patients in carbamazepine group (HR 0.50, 95 % CI 0.16–1.57), valproic acid group (HR 0.64, 95 % CI 0.33–1.25), or phenytoin group (HR 0.95, 95 % CI 0.55–1.65), compared to those in the new ASM group. (Table 3) The Kaplan–Meyer curves of survival rate and new ischemic stroke rate in the four groups were shown in Fig. 2. A similar trend was found in the “first ASM model” (shown in Supplementary Table 1). The risk of the 5-year mortality was also higher in phenytoin group (HR 1.44, 95 % CI 1.20–1.71) compared to new ASM group.

**Table 2:** Death and new ischemic stroke in the four ASM groups

	Phenytoin (n = 3917)	Valproic acid (n = 1623)	Carbamazepine (n = 457)	New ASM (n = 965)	*P* value
Mortality in the follow-up period, n (%)	237(6.1 %)	56(3.5 %)	4(0.9 %)	23(2.4 %)	< 0.0001
Interval from index date of ASM use to death, years, mean (SD)	0.9(1.1)	0.8(0.8)	1.3(1.7)	0.8(1.0)	0.5362
New ischemic stroke in the follow-up period, n (%)	83(2.1 %)	25(1.5 %)	4(0.9 %)	20(2.1 %)	< 0.0001
Interval from index date of ASM use to new ischemic stroke, years, mean (SD)	1.6(1.6)	1.4(1.2)	0.9(0.6)	1.4(1.4)	0.7513

Abbreviations: *ASM* anti-seizure medication; *SD* standard deviation

**Table 3:** The risk of 5-year mortality and new ischemic strokes in the four ASM groups

Outcome	Crude HR (95 % CI)	*P* value	Adjusted HR^a^ (95 % CI)	*P* value
**Follow-up for 5 years**				
**Death**				
New ASM	Reference		Reference	
Carbamazepine	0.40(0.14–1.16)	0.09	0.47(0.14–1.62)	0.23
Valproic acid	1.35(0.83–2.19)	0.23	1.03(0.62–1.70)	0.92
Phenytoin	1.95(1.27-3.00)	0.002	1.64(1.06–2.55)	0.03
**New ischemic strokes (competing risk analysis)**				
New ASM	Reference		Reference	
Carbamazepine	0.45(0.15–1.32)	0.15	0.50(0.16–1.57)	0.23
Valproic acid	0.68(0.38–1.22)	0.20	0.64(0.33–1.25)	0.19
Phenytoin	0.81(0.50–1.34)	0.42	0.95(0.55–1.65)	0.87

Abbreviations: *ASM* anti-seizure medication, *HR* hazard ratio, *CI* confidence interval

^**a**^Adjusted for age, gender, index stroke type, hypertension, diabetic mellitus, hyperlipidemia, ischemic heart disease, heart failure, atrial fibrillation, prior stroke, chronic kidney disease, peripheral vascular disease, mood disorder, dementia, migraine, stroke severity index, concomitant anticoagulants use, concomitant antiplatelet use, concomitant antidepressants use, concomitant statin use, and Year of index date of ASM use

The subgroup analyses suggested phenytoin compared with new ASMs was associated with an increased risk of 5-year mortality in patients with PSE with hyperlipidemia but not patients with PSE without hyperlipidemia (hyperlipidemia: HR 2.76, 95 % CI 1.37–5.56 vs. no hyperlipidemia: HR 1.11, 95 % CI 0.63–1.96, P for interaction = 0.04) (Table 4).

**Table 4:** Subgroups analysis in the phenytoin vs. the New ASM groups

	Adjusted HR (95 % CI)*	P for interaction
Sex		0.41
Male	1.98(1.05–3.74)	
Female	1.35(0.73–2.49)	
Index stroke type		0.41
Ischemic stroke	1.52(0.95–2.45)	
Hemorrhagic stroke	2.42(0.74–7.97)	
Hyperlipidemia		0.04
Yes	2.76(1.37–5.56)	
No	1.11(0.63–1.96)	
Ischemic heart disease		0.79
Yes	1.59(0.90–2.82)	
No	1.84(0.92–3.70)	
Heart failure		0.83
Yes	2.04(0.73–5.68)	
No	1.70(1.03–2.78)	
Atrial fibrillation		0.75
Yes	1.35(0.60–3.06)	
No	1.69(1.00-2.86)	
Mood disorder		0.43
Yes	6.33(0.50–79.90)	
No	1.57(1.00-2.46)	
Dementia		0.18
Yes	1.15(0.52–2.53)	
No	1.95(1.14–3.33)	
Migraine		0.96
Yes	-	
No	1.62(1.03–2.53)	
Stroke severity index		0.93
Mild	1.70(0.49–5.85)	
Moderate	1.86(1.05–3.30)	
Severe	1.15(0.48–2.77)	
Concomitant anticoagulants use		0.97
Yes	-	
No	1.39(0.90–2.17)	
Concomitant antidepressants use		0.39
Yes	6.39(0.73–55.56)	
No	1.55(0.99–2.43)	
Concomitant antiplatelet use		0.55
Yes	1.54(0.85–2.77)	
No	1.66(0.85–3.24)	
Concomitant Statin use		0.09
Yes	8.07(0.98–66.76)	
No	1.38(0.88–2.17)	
Medication use duration		0.34
< 90 days	2.63(1.25–5.52)	
90–179 days	1.58(0.60–4.16)	
≥ 180 days	1.32(0.68–2.56)	
Year of index date of ASM use		0.96
2001–2007	1.83(0.66–5.05)	
2008–2013	1.92(1.18–3.13)	

Abbreviations: *ASM* anti-seizure medication, *HR* hazard ratio, *CI* confidence interval

The average daily dose of each ASM in all patients and in those who had mortality within 5 years in each group were summarized in Supplementary Table 2. There was no difference in the average daily dose of each ASM in all patients and in those who died within 5 years.

## Discussion

To our knowledge, this is the first study investigating the long-term effect of ASM monotherapy on the risk of all-cause death and new ischemic stroke in patients with PSE. There is a paucity of studies about the risk of death or vascular events associated with ASM use [9, 19, 20]. Studies have found that ASM-treated epilepsy was associated with a higher risk of myocardial infarction, stroke, cardiovascular death, all-cause death and sudden cardiac death [9, 20]. A study revealed that ASM use was associated with an increased risk of death in patients with Alzheimer disease [19]. It has been reported that older ASMs, such as phenytoin, carbamazepine, valproic acid, or phenobarbital, were linked to a higher risk of death or stroke in patients with epilepsy without prior stroke [9, 10, 19]. However, older ASMs are still frequently prescribed as the first-line therapy in patients with PSE. In our cohort, 86 % of patients with PSE used older ASMs as the first-line monotherapy.

There are some potential explanations for our finding that long-term phenytoin monotherapy was associated with a higher death risk in patients with PSE. A Sweden study found that the disorders of the circulatory systems were the most common cause of death in the patients with PSE [21]. Therefore, the increased death risk in phenytoin user may be contributed from its influences on the circulatory systems. Phenytoin was associated with a higher risk of hyperlipidemia [22, 23], increased homocysteine and high sensitivity C-reactive protein [24], and increased atherosclerosis measured as intima media thickness [24]. Phenytoin is a sodium channel blocker. Sodium channel blocking ASMs were reported to be related to a higher risk of sudden cardiac death and arrhythmogenic ST-T abnormality [20, 25]. Decreased cardiac sodium current may increase the risk of ventricular fibrillation and sudden cardiac death [20]. Cases of arrhythmia and sinus arrest has also been reported in phenytoin users [26]. Phenytoin has been shown to inhibit the IKr and a possible role in sudden unexpected death in epilepsy patients (SUDEP) has been suggested [27]. Furthermore, phenytoin has a zero-order metabolism and may easily result in overdose that may present as arrhythmia, respiratory arrest or sinus arrest. Finally, a study had revealed that patients with PSE using phenytoin had a higher risk of emergency room visits or hospitalization due to seizures, compared to the patients with PSE using new ASMs [28]. Poor control of seizures may also increase the risk of seizure-related death and SUDEP [28].

On the other hand, we did not find a difference in new ischemic stroke risk across the various ASMs groups in patients with PSE. But phenytoin has been reported to be related to a higher stroke risk in patients with epilepsy without stroke history [10]. We thought that physicians might pay more attention to monitor and to treat the vascular risk factors in patients with PSE. These stroke prevention strategies may mask the small effects of ASMs on new ischemic stroke risk.

Our study showed that the ischemic stroke and mortality rate were lowest in carbamazepine group. There might be some bias in this finding because many patients were censored from the carbamazepine group due to medication withdrawal before any study outcome happened. At recruitment, there were 457 patients in carbamazepine group, but at the end of the 1st year, there were only 76 patients remained. Carbamazepine is notorious in its risk of severe hypersensitivity reactions in Asians [29], so physicians may tend to withdrawal it if the patients reported any discomforts.

The main strength of our study was that it was based on a nationwide database, that provided advantages of large case numbers, long follow-up duration, complete data of the duration and the dosage of ASMs, and less selection bias related to socioeconomic status, job situation or different healthcare institutions. Although the diagnosis of stroke and epilepsy could not be confirmed by medical chart review, the two diagnoses had been validated in NHIRD, with an accuracy of 94 % for stroke and 99.83 % for epilepsy [14, 16]. Another strength of our study was that we focused on first-line ASM monotherapy. Previous studies showed that more than 70 % of the patients with PSE could achieve seizure freedom under ASM monotherapy [5], and the 5-year retention rate of first ASM in patients with PSE was high (55–75 %) [30]. Therefore, selection of first ASM in patients with PSE is important in clinical practice, and our study provided an important reference on this issue.

This study has limitations. First, some important information, such as the types and the severity of the seizures, reasons to choose or not to choose a specific ASM, the patients’ laboratory data and imaging findings, and the TOAST classification of the index stroke, could not be assessed by NHIRD. Second, we did not know the drug compliance and the serum concentration of the ASMs in each patient. Inadequate dosage of ASMs may result in poor seizures control or more severe side effects. Third, we could not ascertain the cause of death from our database, and we could not evaluate if there were any differences in the cause of death in different ASM groups. Fourth, although our study design included adequate control of numerous confounding factors, unmeasured or unknown confounders may have generated a bias. For example, we were not able to account for effects of smoking, physical activity, alcohol intake, diet habits, a family history of cardiovascular diseases, body mass index or socioeconomical status. Fifth, our study included the patients with PSE who had no switches of their ASM. These patients may be younger, with fewer comorbidities, and with lesser disease severity. Therefore, our study population may not be representative of the whole population of PSE. Finally, this study used national health insurance database in Taiwan, and most insured people in this insurance system are Asians. Therefore, the result of the study may not be generalizable to other populations in different races or countries. The generalizability of the current results needs to be confirmed by further studies conducted in other countries and populations.

In conclusion, our study showed that using phenytoin as the first-line ASM monotherapy, compared with using new ASMs, was associated with an increase in 5-year death risk among patients with PSE. This finding provides potentially vital information for clinicians to improve the long-term survival of patients with PSE. Still, prospectively designed cohort studies and randomized controlled trials are warranted, to identify the most optimal ASM regimen(s) for patients with PSE.

## Supplementary Information

Additional file 1

Additional file 2

## Data Availability

The datasets generated and/or analysed during the current study are not publicly available because this was a national insurance database but are available from the corresponding author on reasonable request.
