# Supplementary material for: Effects of long-term anti-seizure medication monotherapy on all-cause death in patients with post-stroke epilepsy: a nationwide population-based study in Taiwan
Source: BMC Neurol. 2021 Jun 21;21:226. doi: 10.1186/s12883-021-02241-5 (PMC8215791; doi:10.1186/s12883-021-02241-5)
Supplement: Supplementary file 1 — Additional file 1 [file 12883_2021_2241_MOESM1_ESM.doc]

**Title page**

# Title: Effects of long-term Anti-Seizure Medication Monotherapy on All-cause Death in Patients with Post-Stroke Epilepsy: A Nationwide Population-based Study in Taiwan

**Cover title: Anti-seizure medication for post-stroke epilepsy**

Chia-Yu Hsu, MD^1^; Chun-Yu Cheng, MD^2^; Jiann-Der Lee, MD PhD^1^; Meng Lee, MD^1^; Bruce Ovbiagele, MD FRCP^3^

^1^Departments of Neurology, Chang Gung University College of Medicine, Chang Gung Memorial Hospital, Chiayi, Taiwan; ^2^Departments of Neurosurgery, Chang Gung University College of Medicine, Chang Gung Memorial Hospital, Chiayi, Taiwan; ^3^Department of Neurology, University of California, San Francisco, California, USA

Supplementary Table 1 Sensitivity analysis of the risk of 5-year mortality and new ischemic strokes in the four ASM groups

| Outcome | Crude HR  (95% CI) | P value | Adjusted HR*  (95% CI) | P value |
| --- | --- | --- | --- | --- |
| **Sensitivity analysis: First ASM model (follow-up for 5 years)** | | | | |
| Death |  |  |  |  |
| New ASM | Reference |  | Reference |  |
| Carbamazepine | 0.82(0.62-1.07) | 0.14 | 0.89(0.66-1.20) | 0.44 |
| Valproic acid | 1.62(1.34-1.95) | <0.001 | 1.47(1.22-1.79) | <0.001 |
| Phenytoin | 1.53(1.29-1.82) | <0.001 | 1.44(1.20-1.71) | <0.001 |
| Recurrent strokes (competing risk analysis) | | | | |
| New ASM | Reference |  | Reference |  |
| Carbamazepine | 0.84(0.58-1.20) | 0.34 | 0.88(0.60-1.31) | 0.54 |
| Valproic acid | 0.86(0.66-1.14) | 0.29 | 0.90(0.66-1.21) | 0.48 |
| Phenytoin | 0.88(0.69-1.11) | 0.28 | 1.01(0.78-1.31) | 0.94 |

Abbreviations: ASM: anti-seizure medication, HR: hazard ratio, CI: confidence interval

*Adjusted for age, gender, index stroke type, hypertension, diabetic mellitus, hyperlipidemia, ischemic heart disease, heart failure, atrial fibrillation, prior stroke, chronic kidney disease, peripheral vascular disease, mood disorder, dementia, migraine, stroke severity index, concomitant anticoagulants use, concomitant antiplatelet use, concomitant antidepressants use, concomitant statin use, and Year of index date of ASM use
