# Supplementary material for: Effects of long-term anti-seizure medication monotherapy on all-cause death in patients with post-stroke epilepsy: a nationwide population-based study in Taiwan
Source: BMC Neurol. 2021 Jun 21;21:226. doi: 10.1186/s12883-021-02241-5 (PMC8215791; doi:10.1186/s12883-021-02241-5)
Supplement: Supplementary file 2 — Additional file 2 [file 12883_2021_2241_MOESM2_ESM.doc]

**Title page**

# Title: Effects of long-term Anti-Seizure Medication Monotherapy on All-cause Death in Patients with Post-Stroke Epilepsy: A Nationwide Population-based Study in Taiwan

**Cover title: Anti-seizure medication for post-stroke epilepsy**

Chia-Yu Hsu, MD^1^; Chun-Yu Cheng, MD^2^; Jiann-Der Lee, MD PhD^1^; Meng Lee, MD^1^; Bruce Ovbiagele, MD FRCP^3^

^1^Departments of Neurology, Chang Gung University College of Medicine, Chang Gung Memorial Hospital, Chiayi, Taiwan; ^2^Departments of Neurosurgery, Chang Gung University College of Medicine, Chang Gung Memorial Hospital, Chiayi, Taiwan; ^3^Department of Neurology, University of California, San Francisco, California, USA

Supplemental Table 2. Average dose of anti-seizure medications in each group

| Anti-seizure medications | Average daily dose in all included patients (mg/day) | Average daily dosage in patients who had mortality in the follow-up period (mg/day) |
| --- | --- | --- |
| Phenytoin | 276.5 | 276.4 |
| Valproic acid | 774.7 | 781.0 |
| Carbamazepine | 348.9 | 385.6 |
| New anti-seizure medications |  |  |
| gabapentin | 480.1 | 463.6 |
| levetiracetam | 998.5 | 1025.4 |
| oxcarbazepine | 523.5 | 492.7 |
| topiramate | 88.3 | 83.9 |
| lamotrigine | 88.0 | 100.9 |
| clobazem | 12.9 | _ |
| vigabatrin | 893.0 |  |
| pregabalin | 112.4 | _ |
